# Supplementary material for: HIVprotI: an integrated web based platform for prediction and design of HIV proteins inhibitors
Source: J Cheminform. 2018 Mar 9;10:12. doi: 10.1186/s13321-018-0266-y (PMC5845081; doi:10.1186/s13321-018-0266-y)
Supplement: Supplementary file 2 — Additional file 2. Source code of HIVProtI web server. [file 13321_2018_266_MOESM2_ESM.zip › HIVprotI_Source-code/hivprotI/weka-3-6-6/doc/weka/core/NormalizableDistance.html]

NormalizableDistance


---


|  |  |  |
| --- | --- | --- |
| **Overview** \| **Package** \| **Class** \| **Tree** \| **Deprecated** \| **Index** \| **Help** \| **Weka's home** | |  |
| **PREV CLASS**   **NEXT CLASS** | **FRAMES**    **NO FRAMES**     **All Classes** |  |
| SUMMARY: NESTED \| FIELD \| CONSTR \| METHOD | DETAIL: FIELD \| CONSTR \| METHOD |  |


---


## weka.core Class NormalizableDistance

```
java.lang.Object
  weka.core.NormalizableDistance
```

**All Implemented Interfaces:**: java.io.Serializable, DistanceFunction, OptionHandler, RevisionHandler

**Direct Known Subclasses:**: AbstractStringDistanceFunction, ChebyshevDistance, EuclideanDistance, ManhattanDistance

---

``` public abstract class NormalizableDistance extends java.lang.Object implements DistanceFunction, OptionHandler, java.io.Serializable, RevisionHandler ```

Represents the abstract ancestor for normalizable distance functions, like
Euclidean or Manhattan distance.

**Version:**
:   $Revision: 1.2 $

**Author:**
:   Fracpete (fracpete at waikato dot ac dot nz), Gabi Schmidberger (gabi@cs.waikato.ac.nz) -- original code from weka.core.EuclideanDistance, Ashraf M. Kibriya (amk14@cs.waikato.ac.nz) -- original code from weka.core.EuclideanDistance

**See Also:**: Serialized Form

---

| **Field Summary** | |
| --- | --- |
| `static int` | `R_MAX`             Index in ranges for MAX. |
| `static int` | `R_MIN`             Index in ranges for MIN. |
| `static int` | `R_WIDTH`             Index in ranges for WIDTH. |


**Constructor Summary**

`NormalizableDistance()`
  
          Invalidates the distance function, Instances must be still set.

`NormalizableDistance(Instances data)`
  
          Initializes the distance function and automatically initializes the
ranges.


| **Method Summary** | |
| --- | --- |
| `java.lang.String` | `attributeIndicesTipText()`             Returns the tip text for this property. |
| `double` | `distance(Instance first, Instance second)`             Calculates the distance between two instances. |
| `double` | `distance(Instance first, Instance second, double cutOffValue)`             Calculates the distance between two instances. |
| `double` | `distance(Instance first, Instance second, double cutOffValue, PerformanceStats stats)`             Calculates the distance between two instances. |
| `double` | `distance(Instance first, Instance second, PerformanceStats stats)`             Calculates the distance between two instances. |
| `java.lang.String` | `dontNormalizeTipText()`             Returns the tip text for this property. |
| `java.lang.String` | `getAttributeIndices()`             Gets the range of attributes used in the calculation of the distance. |
| `boolean` | `getDontNormalize()`             Gets whether if the attribute values are to be normazlied in distance calculation. |
| `Instances` | `getInstances()`             returns the instances currently set. |
| `boolean` | `getInvertSelection()`             Gets whether the matching sense of attribute indices is inverted or not. |
| `java.lang.String[]` | `getOptions()`             Gets the current settings. |
| `double[][]` | `getRanges()`             Method to get the ranges. |
| `abstract  java.lang.String` | `globalInfo()`             Returns a string describing this object. |
| `double[][]` | `initializeRanges()`             Initializes the ranges using all instances of the dataset. |
| `double[][]` | `initializeRanges(int[] instList)`             Initializes the ranges of a subset of the instances of this dataset. |
| `double[][]` | `initializeRanges(int[] instList, int startIdx, int endIdx)`             Initializes the ranges of a subset of the instances of this dataset. |
| `void` | `initializeRangesEmpty(int numAtt, double[][] ranges)`             Used to initialize the ranges. |
| `boolean` | `inRanges(Instance instance, double[][] ranges)`             Test if an instance is within the given ranges. |
| `java.lang.String` | `invertSelectionTipText()`             Returns the tip text for this property. |
| `java.util.Enumeration` | `listOptions()`             Returns an enumeration describing the available options. |
| `void` | `postProcessDistances(double[] distances)`             Does nothing, derived classes may override it though. |
| `boolean` | `rangesSet()`             Check if ranges are set. |
| `void` | `setAttributeIndices(java.lang.String value)`             Sets the range of attributes to use in the calculation of the distance. |
| `void` | `setDontNormalize(boolean dontNormalize)`             Sets whether if the attribute values are to be normalized in distance calculation. |
| `void` | `setInstances(Instances insts)`             Sets the instances. |
| `void` | `setInvertSelection(boolean value)`             Sets whether the matching sense of attribute indices is inverted or not. |
| `void` | `setOptions(java.lang.String[] options)`             Parses a given list of options. |
| `java.lang.String` | `toString()`             Returns an empty string. |
| `void` | `update(Instance ins)`             Update the distance function (if necessary) for the newly added instance. |
| `void` | `updateRanges(Instance instance)`             Update the ranges if a new instance comes. |
| `double[][]` | `updateRanges(Instance instance, double[][] ranges)`             Updates the ranges given a new instance. |
| `void` | `updateRanges(Instance instance, int numAtt, double[][] ranges)`             Updates the minimum and maximum and width values for all the attributes based on a new instance. |
| `void` | `updateRangesFirst(Instance instance, int numAtt, double[][] ranges)`             Used to initialize the ranges. |

**Methods inherited from class java.lang.Object**

`equals, getClass, hashCode, notify, notifyAll, wait, wait, wait`

**Methods inherited from interface weka.core.RevisionHandler**

`getRevision`

**Field Detail**

### R\_MIN

```
public static final int R_MIN
```

:   Index in ranges for MIN.

    **See Also:**: Constant Field Values

---


### R\_MAX

```
public static final int R_MAX
```

:   Index in ranges for MAX.

    **See Also:**: Constant Field Values

---


### R\_WIDTH

```
public static final int R_WIDTH
```

:   Index in ranges for WIDTH.

    **See Also:**: Constant Field Values


**Constructor Detail**

### NormalizableDistance

```
public NormalizableDistance()
```

:   Invalidates the distance function, Instances must be still set.

---


### NormalizableDistance

```
public NormalizableDistance(Instances data)
```

:   Initializes the distance function and automatically initializes the
    ranges.

    **Parameters:**: `data` - the instances the distance function should work on


**Method Detail**

### globalInfo

```
public abstract java.lang.String globalInfo()
```

:   Returns a string describing this object.

    :   **Returns:**: a description of the evaluator suitable for displaying in the explorer/experimenter gui

---


### listOptions

```
public java.util.Enumeration listOptions()
```

:   Returns an enumeration describing the available options.

    :   **Specified by:**: `listOptions` in interface `OptionHandler`
    :   **Returns:**: an enumeration of all the available options.

---


### getOptions

```
public java.lang.String[] getOptions()
```

:   Gets the current settings. Returns empty array.

    :   **Specified by:**: `getOptions` in interface `OptionHandler`
    :   **Returns:**: an array of strings suitable for passing to setOptions()

---


### setOptions

```
public void setOptions(java.lang.String[] options)
                throws java.lang.Exception
```

:   Parses a given list of options.

    :   **Specified by:**: `setOptions` in interface `OptionHandler`
    :   **Parameters:**: `options` - the list of options as an array of strings **Throws:**: `java.lang.Exception` - if an option is not supported

---


### dontNormalizeTipText

```
public java.lang.String dontNormalizeTipText()
```

:   Returns the tip text for this property.

    :   **Returns:**: tip text for this property suitable for displaying in the explorer/experimenter gui

---


### setDontNormalize

```
public void setDontNormalize(boolean dontNormalize)
```

:   Sets whether if the attribute values are to be normalized in distance
    calculation.

    :   **Parameters:**: `dontNormalize` - if true the values are not normalized

---


### getDontNormalize

```
public boolean getDontNormalize()
```

:   Gets whether if the attribute values are to be normazlied in distance
    calculation. (default false i.e. attribute values are normalized.)

    :   **Returns:**: false if values get normalized

---


### attributeIndicesTipText

```
public java.lang.String attributeIndicesTipText()
```

:   Returns the tip text for this property.

    :   **Returns:**: tip text for this property suitable for displaying in the explorer/experimenter gui

---


### setAttributeIndices

```
public void setAttributeIndices(java.lang.String value)
```

:   Sets the range of attributes to use in the calculation of the distance.
    The indices start from 1, 'first' and 'last' are valid as well.
    E.g.: first-3,5,6-last

    :   **Specified by:**: `setAttributeIndices` in interface `DistanceFunction`
    :   **Parameters:**: `value` - the new attribute index range

---


### getAttributeIndices

```
public java.lang.String getAttributeIndices()
```

:   Gets the range of attributes used in the calculation of the distance.

    :   **Specified by:**: `getAttributeIndices` in interface `DistanceFunction`
    :   **Returns:**: the attribute index range

---


### invertSelectionTipText

```
public java.lang.String invertSelectionTipText()
```

:   Returns the tip text for this property.

    :   **Returns:**: tip text for this property suitable for displaying in the explorer/experimenter gui

---


### setInvertSelection

```
public void setInvertSelection(boolean value)
```

:   Sets whether the matching sense of attribute indices is inverted or not.

    :   **Specified by:**: `setInvertSelection` in interface `DistanceFunction`
    :   **Parameters:**: `value` - if true the matching sense is inverted

---


### getInvertSelection

```
public boolean getInvertSelection()
```

:   Gets whether the matching sense of attribute indices is inverted or not.

    :   **Specified by:**: `getInvertSelection` in interface `DistanceFunction`
    :   **Returns:**: true if the matching sense is inverted

---


### setInstances

```
public void setInstances(Instances insts)
```

:   Sets the instances.

    :   **Specified by:**: `setInstances` in interface `DistanceFunction`
    :   **Parameters:**: `insts` - the instances to use

---


### getInstances

```
public Instances getInstances()
```

:   returns the instances currently set.

    :   **Specified by:**: `getInstances` in interface `DistanceFunction`
    :   **Returns:**: the current instances

---


### postProcessDistances

```
public void postProcessDistances(double[] distances)
```

:   Does nothing, derived classes may override it though.

    :   **Specified by:**: `postProcessDistances` in interface `DistanceFunction`
    :   **Parameters:**: `distances` - the distances to post-process

---


### update

```
public void update(Instance ins)
```

:   Update the distance function (if necessary) for the newly added instance.

    :   **Specified by:**: `update` in interface `DistanceFunction`
    :   **Parameters:**: `ins` - the instance to add

---


### distance

```
public double distance(Instance first,
                       Instance second)
```

:   Calculates the distance between two instances.

    :   **Specified by:**: `distance` in interface `DistanceFunction`
    :   **Parameters:**: `first` - the first instance: `second` - the second instance **Returns:**: the distance between the two given instances

---


### distance

```
public double distance(Instance first,
                       Instance second,
                       PerformanceStats stats)
```

:   Calculates the distance between two instances.

    :   **Specified by:**: `distance` in interface `DistanceFunction`
    :   **Parameters:**: `first` - the first instance: `second` - the second instance: `stats` - the performance stats object **Returns:**: the distance between the two given instances

---


### distance

```
public double distance(Instance first,
                       Instance second,
                       double cutOffValue)
```

:   Calculates the distance between two instances. Offers speed up (if the
    distance function class in use supports it) in nearest neighbour search by
    taking into account the cutOff or maximum distance. Depending on the
    distance function class, post processing of the distances by
    postProcessDistances(double []) may be required if this function is used.

    :   **Specified by:**: `distance` in interface `DistanceFunction`
    :   **Parameters:**: `first` - the first instance: `second` - the second instance: `cutOffValue` - If the distance being calculated becomes larger than cutOffValue then the rest of the calculation is discarded. **Returns:**: the distance between the two given instances or Double.POSITIVE\_INFINITY if the distance being calculated becomes larger than cutOffValue.

---


### distance

```
public double distance(Instance first,
                       Instance second,
                       double cutOffValue,
                       PerformanceStats stats)
```

:   Calculates the distance between two instances. Offers speed up (if the
    distance function class in use supports it) in nearest neighbour search by
    taking into account the cutOff or maximum distance. Depending on the
    distance function class, post processing of the distances by
    postProcessDistances(double []) may be required if this function is used.

    :   **Specified by:**: `distance` in interface `DistanceFunction`
    :   **Parameters:**: `first` - the first instance: `second` - the second instance: `cutOffValue` - If the distance being calculated becomes larger than cutOffValue then the rest of the calculation is discarded.: `stats` - the performance stats object **Returns:**: the distance between the two given instances or Double.POSITIVE\_INFINITY if the distance being calculated becomes larger than cutOffValue.

---


### initializeRanges

```
public double[][] initializeRanges()
```

:   Initializes the ranges using all instances of the dataset.
    Sets m\_Ranges.

    :   **Returns:**: the ranges

---


### updateRangesFirst

```
public void updateRangesFirst(Instance instance,
                              int numAtt,
                              double[][] ranges)
```

:   Used to initialize the ranges. For this the values of the first
    instance is used to save time.
    Sets low and high to the values of the first instance and
    width to zero.

    :   **Parameters:**: `instance` - the new instance: `numAtt` - number of attributes in the model: `ranges` - low, high and width values for all attributes

---


### updateRanges

```
public void updateRanges(Instance instance,
                         int numAtt,
                         double[][] ranges)
```

:   Updates the minimum and maximum and width values for all the attributes
    based on a new instance.

    :   **Parameters:**: `instance` - the new instance: `numAtt` - number of attributes in the model: `ranges` - low, high and width values for all attributes

---


### initializeRangesEmpty

```
public void initializeRangesEmpty(int numAtt,
                                  double[][] ranges)
```

:   Used to initialize the ranges.

    :   **Parameters:**: `numAtt` - number of attributes in the model: `ranges` - low, high and width values for all attributes

---


### updateRanges

```
public double[][] updateRanges(Instance instance,
                               double[][] ranges)
```

:   Updates the ranges given a new instance.

    :   **Parameters:**: `instance` - the new instance: `ranges` - low, high and width values for all attributes **Returns:**: the updated ranges

---


### initializeRanges

```
public double[][] initializeRanges(int[] instList)
                            throws java.lang.Exception
```

:   Initializes the ranges of a subset of the instances of this dataset.
    Therefore m\_Ranges is not set.

    :   **Parameters:**: `instList` - list of indexes of the subset **Returns:**: the ranges **Throws:**: `java.lang.Exception` - if something goes wrong

---


### initializeRanges

```
public double[][] initializeRanges(int[] instList,
                                   int startIdx,
                                   int endIdx)
                            throws java.lang.Exception
```

:   Initializes the ranges of a subset of the instances of this dataset.
    Therefore m\_Ranges is not set.
    The caller of this method should ensure that the supplied start and end
    indices are valid (start <= end, end<instList.length etc) and
    correct.

    :   **Parameters:**: `instList` - list of indexes of the instances: `startIdx` - start index of the subset of instances in the indices array: `endIdx` - end index of the subset of instances in the indices array **Returns:**: the ranges **Throws:**: `java.lang.Exception` - if something goes wrong

---


### updateRanges

```
public void updateRanges(Instance instance)
```

:   Update the ranges if a new instance comes.

    :   **Parameters:**: `instance` - the new instance

---


### inRanges

```
public boolean inRanges(Instance instance,
                        double[][] ranges)
```

:   Test if an instance is within the given ranges.

    :   **Parameters:**: `instance` - the instance: `ranges` - the ranges the instance is tested to be in **Returns:**: true if instance is within the ranges

---


### rangesSet

```
public boolean rangesSet()
```

:   Check if ranges are set.

    :   **Returns:**: true if ranges are set

---


### getRanges

```
public double[][] getRanges()
                     throws java.lang.Exception
```

:   Method to get the ranges.

    :   **Returns:**: the ranges **Throws:**: `java.lang.Exception` - if no randes are set yet

---


### toString

```
public java.lang.String toString()
```

:   Returns an empty string.

    :   **Overrides:**: `toString` in class `java.lang.Object`
    :   **Returns:**: an empty string


---


|  |  |  |
| --- | --- | --- |
| **Overview** \| **Package** \| **Class** \| **Tree** \| **Deprecated** \| **Index** \| **Help** \| **Weka's home** | |  |
| **PREV CLASS**   **NEXT CLASS** | **FRAMES**    **NO FRAMES**     **All Classes** |  |
| SUMMARY: NESTED \| FIELD \| CONSTR \| METHOD | DETAIL: FIELD \| CONSTR \| METHOD |  |


---
